# Exploring the Influencing Factors of Professional Identity in Geriatric Nursing Among Undergraduate Nursing Interns From a Life Course Perspective: A Qualitative Study

**DOI:** 10.1155/jonm/5532147

**Published:** 2026-02-02

**Authors:** Yiping Chen, Peijing Hao, Yan Lou, Xinyi Han

**Affiliations:** ^1^ School of Nursing, Beijing University of Chinese Medicine, Beijing, China, bucm.edu.cn; ^2^ School of Nursing, Zhejiang Haining Health School, Haining, Zhejiang, China; ^3^ School of Nursing, Hangzhou Normal University, Hangzhou, Zhejiang, China, hznu.edu.cn

**Keywords:** geriatric nursing, nursing education, professional identity, qualitative study

## Abstract

**Background:**

With the intensifying global trend of population aging, the importance of geriatric nursing is becoming increasingly prominent. Understanding how nursing students develop professional identity within the context of geriatric care can help foster their interest and competence, thereby ensuring the quality of care services in an aging society.

**Aim:**

This study aims to explore, from a life course perspective, the multiple factors that influence the formation of professional identity in geriatric nursing among nursing students.

**Methods:**

An exploratory descriptive qualitative design was employed. Semistructured interviews were conducted with 16 undergraduate nursing students. Thematic analysis was used to identify key themes related to the formation of professional identity.

**Results:**

Four main themes emerged from the data: (1) Early life experiences shaping emotional connection and basic impressions of older adults, (2) cognitive construction and challenges in geriatric nursing during education, (3) professional identity development and obstacles during clinical and volunteer experiences, and (4) realistic considerations and societal influences in career planning.

**Conclusion:**

Nursing students’ professional identity is dynamically constructed through the interplay of multidimensional experiences over the life course. Enhancing early exposure, enriching educational content, strengthening clinical support, and optimizing the broader social environment are crucial to promoting positive identification with geriatric nursing and advancing the development of a specialized workforce in this field.

## 1. Introduction

Population aging has become a global trend, posing profound challenges to healthcare systems worldwide. It is projected that by 2050, around 20% of the global population will be over the age of 65, with more than 80% of older adults residing in low‐ and middle‐income countries [1]. This trend not only increases the burden on healthcare systems but also places greater demands on the nursing workforce. As the backbone of healthcare services, nurses play an irreplaceable role in addressing the complex physical and mental health needs of older adults. However, the number of professional nurses willing to engage in geriatric care remains far below actual demand [2], particularly in low‐ and middle‐income countries. A growing concern is the low interest among nursing students in geriatric care [3], contributing to a structural shortage of talent in this field.

Professional identity formation (PIF) is a critical developmental process in nursing education. It not only shapes nursing students′ understanding and identification with their professional roles but also profoundly influences their career choices, clinical behavior, and interpersonal interactions [4]. PIF refers to the gradual internalization of the values, ethical standards, and behavioral norms of the nursing profession by students through learning and practice, ultimately forming a self‐concept of being a “nurse.” However, the factors influencing PIF are multifaceted, encompassing personal life experiences, educational environments, and sociocultural contexts [5]. In the specific field of geriatric nursing, the formation of professional identity is not only affected by professional knowledge but is also deeply shaped by individuals’ attitudes and values toward aging.

In the context of Asian sociocultural settings, nursing students’ professional identity development follows a unique trajectory. On the one hand, Confucian values emphasize filial piety and family responsibility, framing care for the elderly as a moral obligation rather than a professional choice [6]. On the other hand, implicit societal biases against aging and the stereotypical perception of geriatric care as “low‐value” and “low‐skilled” subtly undermine students’ sense of identification with the profession [7, 8]. Moreover, a study from Macao has shown [9] that nursing students’ preferred career choices are community nursing, pediatric nursing, surgical nursing, and obstetric–gynecological nursing, with geriatric nursing rarely mentioned. Another study conducted in China also indicated [10] that nursing students’ motivation to choose a career in geriatric nursing is at a moderate level, suggesting that this motivation needs to be further enhanced. Therefore, it remains necessary to explore nursing students’ professional identity in geriatric nursing in order to increase the likelihood of their career choice in this field.

While existing research has explored the influence of personal, familial, institutional, and structural factors on nursing students′ professional identity, there is a lack of studies that systematically examine how students form attitudes and values toward geriatric nursing throughout their developmental journey from a life course perspective. Life course theory emphasizes [11] the cumulative and contextual nature of individual experiences, focusing on how key life events, such as close relationships with grandparents, exposure to geriatric nursing courses, or clinical internships, can have lasting impacts on professional attitudes. Although recent qualitative studies have begun to explore these dynamic processes, for example, Bolt et al. [12] revealed lifelong motivational mechanisms behind geriatric nurses’ professional identity, and Moll‐Jongerius et al. [13] highlighted the positive effects of humanistic care experiences during clinical placements on medical students’ identity formation, there remains a research gap in understanding how nursing students in Asian cultural contexts construct their professional identity in geriatric care through their life experiences.

Therefore, this study adopts a life course perspective and employs qualitative research methods to explore how nursing students gradually form their professional identity in geriatric care throughout their development and education. We pay particular attention to how early life experiences, socialization processes, and cultural values shape their understanding and attitudes toward aging and nursing work. By filling the current research gap, this study aims to provide culturally sensitive and individually tailored recommendations for nursing education, thereby contributing to the cultivation of a workforce of geriatric nursing professionals with strong professional identity and cultural adaptability.

## 2. Methods

### 2.1. Study Design

This study adopted an exploratory descriptive qualitative design, integrating a life course perspective to explore how undergraduate nursing interns form their professional identity in the context of geriatric nursing. The study followed the 32‐item Consolidated Criteria for Reporting Qualitative Research (COREQ) checklist to ensure methodological rigor and transparency.

### 2.2. Theoretical Framework

The study was guided by the life course theory, which emphasizes that individual development is the cumulative and dynamic result of interactions among sociocultural, institutional, and personal factors. Key concepts include “transitions,” “trajectories,” “turning points,” and “timing of life events.” This framework supports an understanding of how nursing students’ attitudes and professional identity in geriatric care are gradually shaped and transformed throughout their life experiences and educational journeys.

### 2.3. Research Team and Reflexivity

Interviews were conducted by two female researchers (Authors B and C), both of whom had received qualitative research training. One is a doctoral candidate in nursing with hands‐on experience in geriatric care and qualitative interviewing, while the other is a research assistant in nursing education. A third senior researcher (Author A), a clinical nursing educator, participated in data analysis and review but was not involved in initial coding to minimize role‐related bias. No prior relationship existed between researchers and participants before the study. Participants were informed at the outset that the research team was interested in understanding how their life experiences and education influenced their attitudes and identity formation related to geriatric care. Throughout the study, researchers maintained reflexivity by documenting their assumptions, backgrounds, and potential biases through reflective journals and memos to enhance the credibility of the findings.

### 2.4. Research Setting

The study was conducted at a university in northern China that offers a nationally accredited undergraduate nursing education program. The region is one of the economically developed areas in northern China, characterized by concentrated medical and educational resources and a relatively high level of population aging, with adults aged 60 years and above accounting for more than 20% of the local population. This demographic structure provides nursing students with extensive exposure to older adult patients during both academic and clinical training. The curriculum includes both theoretical instruction and clinical placements, with key internship departments serving a high proportion of older adult patients, such as geriatrics, community health, and internal medicine. This context offers students rich opportunities to engage in geriatric care practices, thereby enhancing the relevance of exploring their PIF in this field.

### 2.5. Participants and Recruitment

A purposive sampling strategy was used to recruit information‐rich participants capable of deep reflection. The inclusion criteria were as follows: (1) full‐time fourth‐year undergraduate nursing students, (2) completion of at least one clinical rotation involving the care of older adults, and (3) willingness to share personal and professional experiences related to geriatric care. Invitation letters were distributed by class advisors and course coordinators. In total, 16 students (13 females and 3 males, aged 21–23) consented to participate in the study. To ensure sample diversity, factors such as gender, clinical department experience, and urban–rural background were considered during recruitment.

### 2.6. Data Collection

Data collection was conducted from October 2024 to April 2025 through semistructured in‐depth interviews. All interviews were held online via Tencent Meeting, a secure video conferencing platform (by Authors B and C). The interview guide was developed based on life course theory and covered topics such as early interactions with older adults, perceptions of aging, educational experiences in geriatric nursing (both classroom and clinical), and future career planning. The guide was reviewed by two qualitative research experts and pilot‐tested with one nursing student, not included in the final sample, to ensure clarity and relevance.

Each interview lasted 45–60 min and was audio‐recorded with participants’ informed consent. Only the participant and the researcher were present during the interviews. All recordings were transcribed verbatim by the researchers and anonymized. Immediately following each interview, the researchers wrote field notes and memos to capture nonverbal cues and preliminary analytic ideas. No repeat interviews were conducted.

### 2.7. Data Analysis and Trustworthiness

Thematic analysis was conducted based on the six‐phase approach proposed by Braun and Clarke (2022). A hybrid strategy combining deductive and inductive methods was applied. Initially, the research team used life course theory concepts such as “turning points” and “trajectories” to structure a preliminary coding framework. At the same time, they remained open to novel themes emerging from the data. Microsoft Excel was used to manage coding and track theme development. Two researchers independently coded the transcripts, and then integrated and refined codes through team discussions. A third researcher reviewed the full coding framework and thematic structure, offering suggestions for revision. Thematic saturation was confirmed after the 14th interview, with no new themes identified in the final two interviews. To enhance trustworthiness, the research team maintained a detailed audit trail including coding logs, reflective notes, and decision memos. Reflexive writing was conducted throughout the analysis to mitigate potential bias. Four participants were invited to review a summary of the thematic structure, and all affirmed that the findings accurately reflected their experiences, completing the process of member checking.

### 2.8. Ethical Considerations

This study was approved by the institutional review board of the university where the research team is based (Approval no. 2024BZYLL0514). All participants received detailed written and verbal explanations of the study purpose, procedures, and confidentiality measures and signed informed consent forms prior to the interviews. Participation was entirely voluntary, and students could withdraw at any point without academic consequences. All identifiable information was removed during transcription, and the data were stored in encrypted files to ensure confidentiality and privacy protection.

## 3. Results

### 3.1. Characteristics of Participants

Sixteen undergraduate nursing students (13 females, 3 males) participated in the study. 62.5% were not only children, and 75.0% had, at some point, lived with their grandparents. Over half reported frequent contact with older adults (62.5%) and came from urban or town areas (68.7%). Half identified as extroverted (50.0%), and most chose nursing voluntarily (68.8%). Nine (56.2%) expressed willingness to work in geriatric care (Table 1).

**Table 1:** Characteristics of participants.

Variable	Category	*n* (%)
Only child	No	10 (62.5)
	Yes	6 (37.5)

Sex	Male	3 (18.8)
	Female	13 (81.2)

Lived with grandparents	Yes, for some time	7 (43.8)
	Yes, for a long time	5 (31.2)
	No	4 (25.0)

Contact frequency with older adults	Several times a week	3 (18.8)
	Once a week	4 (25.0)
	Twice a month	3 (18.8)
	Once a month or less	6 (37.5)

Residence	Urban	6 (37.5)
	Town	5 (31.2)
	Rural	5 (31.2)

Personality	Extroverted	8 (50.0)
	Introverted	7 (43.8)
	Neither	1 (6.2)

Chose nursing voluntarily	Yes	11 (68.8)
	No	5 (31.2)

Family economic status	Average	11 (68.8)
	Good	5 (31.2)

Willing to work in geriatric care	Yes	9 (56.2)
	No	7 (43.8)

### 3.2. Themes

After conducting thematic analysis on interviews with 16 undergraduate nursing interns, this study identified four main themes and ten subthemes that influence the formation of professional identity in geriatric nursing (Table 2): (1) Early life experiences shaping emotional connection and basic impressions of older adults, (2) cognitive construction and challenges in geriatric nursing during education, (3) professional identity development and obstacles during clinical and volunteer experiences, and (4) realistic considerations and societal influences in career planning. These themes intertwine throughout the life course of nursing students, collectively forming their developmental trajectory of professional identity in geriatric nursing.

**Table 2:** Identified themes and subthemes.

Themes	Subthemes
1. Early life experiences shaping emotional connection and basic impressions of older adults	1.1 Intimate experiences of living with grandparents 1.2 Observing the aging process evokes responsibility 1.3 Adjusting personal and societal impressions

2. Cognitive construction and challenges in geriatric nursing during education	2.1 Foundational knowledge in formal courses 2.2 Scenario‐based teaching and case resonance 2.3 Lack of teaching resources and curricular gaps

3. Professional identity development and obstacles during clinical and volunteer experiences	3.1 Positive interactions with patients reinforce identity 3.2 Challenges of nursing frustration and emotional burden 3.3 Influence of role models in clinical teaching

4. Realistic considerations and societal influences in career planning	4.1 Family advice and job stability considerations 4.2 Social prejudice undermines professional identity 4.3 Redefining the value and future of geriatric nursing

### 3.3. Early Life Experiences Shaping Emotional Connection and Basic Impressions of Older Adults

Nursing students’ emotional connection to older adults often originated from their formative life experiences, including close relationships formed through intergenerational cohabitation, a sense of responsibility evoked by witnessing aging, and cognitive adjustments between personal impressions and societal portrayals of older people.

#### 3.3.1. Intimate Experiences of Living With Grandparents

Experiences of being raised by grandparents significantly reduced students’ psychological distance from older adults. Many participants described a natural sense of closeness developed through long‐term cohabitation: “*I think it’s probably influenced by my childhood, being raised by my grandparents. I have a really good impression of older people. When I see older uncles and aunties, I feel warmth and kindness.*” (P01) This intimate relationship was often long‐term, with some students living with their grandparents for over a decade: “*I’ve had rich experiences living with older adults, my grandparents raised me. I’ve been with them since I was one.*” (P02) Long‐term cohabitation directly shaped communication skills, making students feel more at ease when interacting with older patients: “*I lived with my grandparents for many years, so I don’t feel there’s any communication barrier with older adults, it feels natural.*” (P14) Many emphasized the cumulative effect of time: “*I must’ve lived with my grandmother for over ten years, more than half my life.*” (P05)

#### 3.3.2. Observing the Aging Process Evokes Responsibility

Witnessing physical decline in their grandparents triggered a sense of helplessness in some students, motivating them to study nursing to give back to their families: “*My grandmother has heart issues and gets breathless. I watch her closely, afraid she’ll faint. That helplessness made me want to learn nursing.*” (P06) This sense of responsibility often transformed into proactive learning: “*Later, due to my grandmother’s declining health and moving into a care home, I really wanted to learn more about geriatric nursing.*” (P01) Hands‐on caregiving experiences further deepened understanding: “*My grandparents are aging and getting weaker. I want to learn how to care for them properly and provide effective support.*” (P02) Some integrated familial and professional roles: “*My parents and grandparents will age too. Choosing this career will help me care for them in the future.*” (P03)

#### 3.3.3. Adjusting Personal and Societal Impressions

Students’ images of older adults were shaped not only by family but also by media and public stereotypes. When personal impressions conflicted with societal portrayals, they experienced cognitive dissonance: “*Older people in my family are nothing like what I see in the media, some reports show them yelling at nurses. I worry if I can handle it.*” (P13) Some noted the complexity of geriatric care: “*Older patients are harder to care for, more frail, more emotionally sensitive. Young people have these issues too, but it’s rarer.*” (P04) Others expressed polarized impressions: “*My views are formed from interactions with my family elders. It’s either they’re very kind or quite difficult, there’s no in-between.*” (P08)

### 3.4. Cognitive Construction and Challenges in Geriatric Nursing During Education

During their nursing education, students’ understanding and attitudes toward geriatric care were shaped by curricular content, teaching methods, and institutional support. While some courses sparked interest, the lack of systemic learning remained a barrier.

#### 3.4.1. Foundational Knowledge in Formal Courses

Courses such as community nursing and medication safety helped students recognize the specific needs of older adults: “*Our community nursing class emphasized medication safety and geriatric syndromes. It was really practical.*” (P12) Some began seriously considering geriatric care as a future direction: “*I only started thinking about specializing in geriatrics after learning more in school.*” (P01) Course content also heightened interest: “*I didn’t use to focus on older adults, but this course really increased my engagement.*” (P02)

#### 3.4.2. Scenario‐Based Teaching and Case Resonance

Simulated teaching and clinical case sharing allowed students to empathize with older adults through experiential learning: *“We had to simulate caring for a Parkinson’s patient. That’s when I realized how hard life is for older people.*” (P07) Flipped classrooms also enhanced understanding: “*Flipped classrooms make concepts stick better than just listening to a lecture.*” (P02) Case stories from clinical instructors were emotionally impactful: “*We mostly learned about geriatric nursing through clinical teachers’ shared experiences.*” (P04)

#### 3.4.3. Lack of Teaching Resources and Curricular Gaps

Despite some inclusion of geriatric content, students felt it lacked depth and systematization: “*If I had learned geriatric care earlier and more systematically, I wouldn’t have panicked so much during clinical rounds.*” (P06) The limited course scope reduced their confidence: “*We didn’t get much targeted preparation in geriatric care.*” (P01) Students suggested earlier practical exposure: “*Schools and hospitals should collaborate so students can interact with older adults earlier, it’s very inspiring.” (P13) Some felt content lacked specialization: “Courses are general, with little specific emphasis on geriatric nursing.*” (P08)

### 3.5. Professional Identity Development and Challenges in Clinical and Volunteer Experiences

Clinical internships and volunteer work provided students with firsthand experience caring for older adults. Positive interactions reinforced their professional identity, while emotional and physical challenges in clinical settings could lead to confusion or hesitation. Clinical instructors also played a subtle yet pivotal role in shaping students’ attitudes.

#### 3.5.1. Positive Interactions With Patients Reinforce Identity

Receiving appreciation from older patients gave students a strong sense of value in their work: “*An elderly lady once said, “You take care of me so attentively.” That moment made me feel like nursing is truly meaningful.*” (P01) Daily interactions often included emotional communication, helping build trust and emotional bonds: “*Grandpas and grandmas love chatting with us, it shortens the distance and makes them more cooperative.*” (P01) Some students experienced a sense of accomplishment during critical situations: “*One time a grandfather’s oxygen levels dropped. He pulled through, and even though I only assisted, I felt an incredible sense of achievement.*” (P03) Volunteer work in elder‐care facilities also helped build early caregiving experiences and emotional connection: “*We organized activities, took them for walks in the park, it felt warm and fulfilling. They even gave us emotional support.*” (P05)

#### 3.5.2. Challenges of Nursing Frustration and Emotional Burden

Students reported feelings of helplessness when dealing with complex cases such as dementia or nighttime delirium: “*One grandpa had delirium and kept calling for his deceased wife. I had no idea how to comfort him, I felt so powerless.*” (P13) Some struggled with communication and cooperation issues: “*He wouldn’t listen, refused medicine, then even stopped eating. I didn’t know how to handle it at all.*” (P01) Emotional invalidation was another recurring theme: “*Some older patients don’t even see us as nurses, just servants. That really hurts.*” (P07) Physical demands and environmental discomforts also made some students question their long‐term career plans: “*It’s exhausting, especially with chronic conditions and detailed care tasks. It drains your energy.*” (P09)

#### 3.5.3. Influence of Role Models in Clinical Teaching

Clinical instructors’ professionalism had a major impact on students’ attitudes toward geriatric care: “*My instructor patiently explained how to communicate with older patients. She really changed how I see this field.*” (P02) Positive behavior inspired imitation and confidence‐building: “*My teacher was so gentle and patient with the elderly. That’s who I want to become.*” (P01) In contrast, dismissive attitudes from teachers could lead to disengagement: “*Some teachers say, “Don’t worry about that patient.” That kind of attitude affects how we value the work.*” (P02) Recognition from instructors also played a critical role in early confidence: “*The first time I drew blood successfully, my teacher praised me in front of everyone, I’ll never forget that moment.*” (P02)

### 3.6. Realistic Considerations and Societal Influences in Career Planning

Students’ professional identity in geriatric nursing was shaped not only by emotions and education but also by external factors such as family advice, job prospects, and social perceptions. Their commitment fluctuated between instrumental participation and active choice, gradually becoming clearer through personal reflection.

#### 3.6.1. Family Advice and Job Stability Considerations

Some students initially lacked specific interest in geriatrics but were influenced by family recommendations and expectations of job stability: “*My mom said nursing is stable, especially with an aging population, so I thought I’d give it a try and see after graduation.*” (P08) Over time, some shifted from passive acceptance to emotional commitment: “*A friend works in geriatric medicine. It made me think, if my family needs help, I’d be able to offer it.*” (P01) Many recognized the societal need: “*In an aging society, improving older adults’ quality of life raises the whole society’s quality of life.*” (P05)

#### 3.6.2. Social Prejudice Undermines Professional Identity

Despite acknowledging the growing societal need for geriatric care, students reported that multiple types of social prejudice continued to weaken their confidence in pursuing this specialty. These prejudices originated from societal norms, family expectations, and media portrayals, each shaping identity formation in different ways. Broader cultural perceptions that devalue geriatric nursing made students question the legitimacy and status of the specialty. One participant noted, “*People still think nursing, especially elder care, is just serving people. Sometimes I doubt myself too*.” (P11) Family backgrounds and norms also shaped how students evaluated geriatric nursing. Some participants explained that personal family structures affected their attitudes toward care work with older adults: “*Those who didn’t grow up with grandparents might see this career very differently.*” (P03) Social media amplified negative portrayals of older patients, intensifying students’ fear and uncertainty. As one participant shared, “*I saw posts on Xiaohongshu app about older patients being hard to deal with, it’s scary*.” (P16)

#### 3.6.3. Redefining the Value and Future of Geriatric Nursing

Despite challenges, some students developed a renewed appreciation for the value of geriatric nursing after gaining knowledge and experience. They began to view it as a promising and systemic discipline: “*Geriatric nursing is the future. Japan already has dedicated elder-care technologies, we should catch up.*” (P13) The aging trend itself was seen as a driving force toward specialization: “*I think geriatric nursing is a promising direction. An aging society needs it.*” (P15) Improving salaries and training was seen as key to attracting more professionals: “*If hospitals raised the pay for geriatric care, more people would definitely join.*” (P10) “*More training in geriatric nursing would attract more people to this field.*” (P12)

## 4. Discussion

This study examined how undergraduate nursing interns form professional identity in geriatric care from a life course perspective. The findings indicate that professional identity does not arise from a single influence. Instead, it reflects the combined contribution of early socialization, educational experiences, clinical exposure, and sociocultural contexts. This conclusion aligns with life course theory, which proposes that key developmental transitions shape attitudes, values, and career pathways across time [14]. It also corresponds with recent studies showing that nursing professional identity develops through the continuous interaction of personal values, educational structures, and organizational cultures [5, 15].

The results further demonstrate that early experiences with older adults provide an important emotional and cognitive basis for identity formation. Interactions with grandparents or older community members during childhood and adolescence enhance empathy, reduce ageism, and increase familiarity with aging‐related contexts. This pattern is consistent with earlier studies showing that intergenerational contact and caregiving responsibilities strengthen positive attitudes toward aging and increase openness to working with older adults [16, 17]. Research on ageism reduction has also shown that meaningful intergenerational engagement can improve students’ perceptions of older people and promote early readiness for identity formation [18, 19]. Recent work on the identity development of medical and nursing students suggests that early emotional connections alone are insufficient unless they are followed by structured learning and clinical reinforcement [13].

During formal nursing education, students enter a stage in which professional values and cognitive understanding begin to consolidate. The present findings indicate that structured geriatric content, positive aging perspectives, and clear value guidance support a stronger professional identity in geriatric care. This interpretation is in line with previous reviews showing that many nursing curricula provide limited gerontological content, which reduces students’ interest in the field [2, 5]. A notable insight from this study concerns the advantage of scenario‐based teaching over lectures. Simulation, case activities, and narrative pedagogy require students to engage actively with professional roles, integrate emotional experience with clinical reasoning, and reflect systematically on their learning. These processes are known to enhance role clarity, responsibility, and professional commitment, all of which are central to identity development [20, 21]. In contrast, lecture‐centered instruction often emphasizes disease and decline and may reinforce ageist assumptions within healthcare education [22]. International curriculum research supports the idea that longitudinal, person‐centered geriatric content that highlights diversity and resilience is more effective in promoting positive identity formation [23].

Clinical practice in this study showed both positive and challenging effects on identity. Many students reported that building trust with older patients, receiving recognition from mentors or families, and managing complex care strengthened their sense of professional value. Similar results appear in previous studies that identify authentic geriatric encounters as important developmental milestones [20, 24]. At the same time, geriatric care often involves significant emotional labor. Caring for individuals with multimorbidity, cognitive impairment, or end‐of‐life needs may lead to emotional strain. Recent evidence has shown that early clinical exposure increases emotional labor and that students without adequate family or social support experience greater pressure [25]. If clinical teaching lacks appropriate supervision, emotional support, or reflective guidance, students may associate geriatric nursing with exhaustion, high workload, or low status. Findings from experienced geriatric nurses also indicate that professional identity is more vulnerable in settings characterized by staff shortages, role ambiguity, and insufficient recognition [15].

A notable finding of this study is that 43.8% of students remained unwilling to pursue geriatric nursing despite positive learning experiences. Several mechanisms may help explain this persistent reluctance. Geriatric nursing continues to hold low social valuation internationally, and students often view it as demanding, poorly supported, and lacking in career advancement, all of which influence career decisions [3, 26, 27]. Students’ aspirations also tend to reflect societal perceptions of prestige across nursing specialties. Acute care or technologically advanced departments are frequently regarded as more rewarding, while geriatric care is often portrayed as routine work with limited opportunities for skill development. In East Asian cultural contexts, family expectations exert additional influence. Prior studies have shown that many students select or avoid specialties based on perceived family approval rather than personal interest [28]. System‐level challenges, including workforce shortages and limited organizational support, further reinforce concerns about long‐term career sustainability in geriatric nursing [15].

The mechanisms identified in this study also reflect current public health priorities and healthy aging strategies. As health systems shift from disease‐centered models toward frameworks emphasizing functional ability and social participation, the demand for a competent geriatric nursing workforce continues to grow [1]. However, research consistently demonstrates that fragmented curricula, insufficient clinical mentoring, and organizational cultures that reinforce low professional status undermine identity development among nursing students [5]. Addressing these challenges requires coordinated reforms across education, practice, and policy. Educational programs should strengthen longitudinal and scenario‐based geriatric training and incorporate structured reflection focused on identity development. Clinical environments should improve supervision, workload distribution, and opportunities for students to observe advanced geriatric roles. At the policy level, improving remuneration, expanding career advancement pathways, and raising societal awareness of the value of geriatric care are essential steps toward reshaping the field’s professional image and supporting sustainable career trajectories.

## 5. Conclusion

This study, adopting a life course perspective, explored the process of PIF in undergraduate nursing interns within the field of geriatric care. The findings highlight that this process is multidimensional and evolves dynamically over time. Early interactions with older adults establish an emotional foundation for identity formation, while educational experiences further promote cognitive construction. However, issues such as insufficient curriculum structure and inadequate practical preparation remain barriers. Clinical practice and volunteer experiences serve as pivotal moments in transforming knowledge into identity, which can either strengthen a sense of professional value or undermine identity if stress remains unresolved. As students near graduation, familial and societal attitudes toward geriatric care significantly influence their career decisions. Therefore, the formation of professional identity must be supported throughout the entire educational journey and be reinforced by positive social support and cultural recognition.

### 5.1. Limitations

This study was conducted at a university in northern China. Although maximum variation sampling was employed, the specific cultural context, economic characteristics, and curriculum design of this region may limit the generalizability of the findings. The study site is located in an economically developed northern region with concentrated medical and educational resources and a relatively high proportion of older adults. While this context provided nursing students with rich exposure to older patients and supported the relevance of our inquiry, it may also differ from regions with fewer resources or lower levels of population aging. In addition, the rural–urban distribution of participants was not fully balanced, and the single‐institution sample may overlook regional or institutional variations in geriatric care attitudes. Future studies should include samples from multiple geographic regions and diverse sociodemographic backgrounds to enhance external validity. Moreover, longitudinal designs could provide deeper insight into how professional identity evolves throughout students’ educational and career trajectories.

## 6. Implications for Clinical Practice

The major theoretical contribution of this study lies in its integration of personal experiences, educational processes, and social context into a comprehensive framework for understanding PIF from a life course perspective. The findings suggest that identity formation in geriatric nursing is not solely the product of “professional education” but also a continuation of “life experience.” In terms of practice, this study offers four recommendations to promote professional identity among nursing students in geriatric care: (1) foster emotional connections with older adults through curriculum design, such as incorporating grandparent–grandchild interaction stories and aging simulation activities; (2) optimize the geriatric nursing curriculum by increasing investments in scenario‐based teaching and clinical simulations; (3) enhance the professional attitudes and teaching competencies of clinical instructors to create a supportive learning environment; and (4) promote public awareness and positive social recognition of geriatric nursing through education and advocacy, breaking down stereotypes and strengthening the social foundation for professional identity.

## Funding

No funding was received for this study.

## Conflicts of Interest

The authors declare no conflicts of interest.

## Data Availability

The data that support the findings of this study are available on request from the corresponding author. The data are not publicly available due to privacy or ethical restrictions.
